# Multi Locus Variable-Number Tandem Repeat (MLVA) Typing Tools Improved the Surveillance of *Salmonella* Enteritidis: A 6 Years Retrospective Study

**DOI:** 10.1371/journal.pone.0117950

**Published:** 2015-02-18

**Authors:** Sophie Bertrand, Guillaume De Lamine de Bex, Christa Wildemauwe, Octavie Lunguya, Marie France Phoba, Benedikt Ley, Jan Jacobs, Raymond Vanhoof, Wesley Mattheus

**Affiliations:** 1 National Reference Centre for *Salmonellas*, Bacterial Diseases Division, Communicable and Infectious Diseases, Scientific Institute of Public Health, Brussels, Belgium; 2 Department of Clinical Microbiology, National Institute for Biomedical Research, Kinshasa, The Democratic Republic of Congo; 3 Institute of Tropical Medicine Antwerp, Antwerp, Belgium; 4 Department of Microbiology and Immunology, Faculty of Medicine, University of Leuven, Belgium; University Medical Center Groningen, NETHERLANDS

## Abstract

Surveillance of *Salmonella enterica* subsp. *enterica* serovar Enteritidis is generally considered to benefit from molecular techniques like multiple-locus variable-number of tandem repeats analysis (MLVA), which allow early detection and confinement of outbreaks. Here, a surveillance study, including phage typing, antimicrobial susceptibility testing and MLVA on 1,535 *S.* Enteritidis isolates collected between 2007 and 2012, was used to evaluate the added value of MLVA for public health surveillance in Belgium. Phage types PT4, PT8, PT21, PT1, PT6, PT14b, PT28 and PT13 dominate the Belgian *S.* Enteritidis population. The isolates of *S.* Enteritidis were most frequently susceptible to all antibiotics tested. 172 different MLVA profiles were detected, of which 9 frequent profiles included 67.2% of the *S.* Enteritidis population. During a serial passage experiment on selected isolates to investigate the *in vitro* stability of the 5 MLVA loci, no variations over time were observed indicating that the MLVA profiles were stable. The MLVA profile of isolates originating from different outbreaks in the Democratic Republic of the Congo (DRC) between 2010 and 2011 were distinct from any of the MLVA profiles found in Belgian isolates throughout the six year observational period and demonstrates that MLVA improves public health surveillance of *S.* Enteritidis. However, MLVA should be complemented with other subtyping methods when investigating outbreaks is caused by the most common MLVA profile.

## Background

Salmonellosis constitutes an important public health problem throughout the world with approximately 93.8 million illnesses and 155,000 deaths each year [1]. Non-typhoid *Salmonella* infections commonly cause self-limiting gastroenteritis, but severe infections including bacteraemia and meningitis have also been reported.

In Belgium, non-typhoidal salmonellosis is, after campylobacteriosis, the most frequently reported foodborne illness [1].

S*almonella* infections are caused by consumption of contaminated food, person-to-person transmission, waterborne transmission and numerous environmental and animal exposures.

The genus *Salmonella* comprises more than 2,579 serotypes [2]. *S*. Enteritidis emerged in the 80’s in Europe and in America. The serotype is capable of systemic colonization of poultry, consequently leading to a contamination of eggs and poultry meat. With the introduction of a vaccination campaign in layer flocks in 2005, the incidence of human salmonellosis in Belgium drastically decreased [3]. The total number of human laboratory-confirmed infections fell from 15,000 to 10,000 per year in the period 2000–2004 to 3,000 to 5,000 annually since 2005 (http://bacterio.wiv-isp.be) [4].

Surveillance programs that timely detect *Salmonella* spp. contaminations in the entire food chain (animal feed, living animals, slaughterhouses, retail sector and restaurants) together with sanitary measurements are essential to detect and prevent human *Salmonella* infections.

In Belgium, data on human salmonellosis cases are centralized in the National Reference Centre for *Salmonella* and *Shigella* (NRCSS) [4]. In addition, isolates are submitted on a voluntary basis to the NRCSS for serotyping. *Salmonella* isolates from meat and meat products, isolated in the context of the official zoonosis monitoring program of the Federal Agency for the Safety of the Food Chain (FASFC), are also transferred to the NRCSS for serotyping and genotyping. In consequence the NRCSS possesses the most comprehensive and up to date data collection on human salmonellosis and *Salmonella* food chain contamination in Belgium [4]. Typing is a powerful tool to investigate outbreak and to find the sources of the contamination. However because Enteritidis is one of the most genetically homogenous serovar, methods with high discriminatory power are needed [5]. Traditionally *S*. Enteritidis isolates are characterised by phage typing [6]. This technique utilizes the ability of bacteriophages to specifically infect certain strains of *Salmonella*, depending on the molecular characteristics of both phage and phage receptor on the bacterial surface [7]. Phage types are assigned on the basis of the ability of a given phage to lyse the investigated isolate [8]. The advantage of phage typing resides in the simplicity of its implementation, which requires only basic laboratory equipment. Nevertheless, ambiguous lysis reactions are common drawbacks, which can lead to misdiagnosis [9].

Pulse field gel electrophoresis (PFGE) is generally accepted as being the “gold standard” for the genotyping of *S*. Enteritidis. The advantage of PFGE is its relatively good discriminatory power. However, PFGE is time-consuming and laborious to perform which makes it less suitable for typing a large number of isolates. In addition, rigorous standardization of the applied protocols is necessary [9].

More recently, a multilocus variable number of tandem repeat analysis (MLVA) harmonized protocol for subtyping of *S*. Enteritidis has been published [10,11]. This method is based on a fragment size analysis of the number of repeats in the variable number tandem repeats (VNTR) region of the bacterial genome and can be beneficial for tracing possible sources of community cases of salmonellosis.

The objective of the current study was to evaluate the potential of MLVA typing for surveillance and outbreak detection of human *S*. Enteritidis by comparing, within a panel of isolates submitted to NRCSS over the 6-year period 2007–2012, the MLVA profiles with the results of phage typing and antibiotic susceptibility testing and by evaluating the *in vitro* stability of MLVA loci in a serial passage experiment.

## Materials and Methods

### Human isolates

In Belgium, *Salmonella* strains isolated from human patients by peripheral clinical laboratories were transferred to NRCSS for serotyping (approximately 3,000 to 5,000 strains/year between 2007 and 2012 [4]). During the 6-year period from 1 January 2007 to 31 December 2012, the Belgian NRCSS received a total of 21,188 human *Salmonella* samples from Belgian clinical laboratories. From the 4,365 isolates (20.6%) that were serotyped as Enteritidis, a random subset (equally distributed over the years and geographically) of 1,498 isolates (34.3%) were analysed by phage typing, antimicrobial susceptibility testing and MLVA.

In addition, 37 isolates (2,4%) collected during outbreaks in Congo between 2010 and 2011 ([12] were also included in this study, resulting in a panel of 1,535 isolates.

### *Salmonella* serotyping and Phage typing

Serotyping of *Salmonella* isolates was performed by slide agglutination with commercial antisera following the Kauffmann-White scheme [2]. Phage typing of *S*. Enteritidis was carried out according to the recommendations of the U.K. Health Protection Agency (Colindale, United Kingdom) [13]. A frequent phage type (PT) was defined as a phage type that was detected in at least 50 isolates during the 6-year period 2007–2012.

### Antimicrobial susceptibility testing

The susceptibility to 13 antibiotics was determined by the disk diffusion (Kirby-Bauer) method following recommendations of the European Committee on Antimicrobial Susceptibility Testing (EUCAST) and using Bio-Rad (Nazareth, Belgium) disks [16]. Inhibition zones were interpreted according to EUCAST criteria [14] for ampicillin (Amp), amoxicillin plus clavulanic acid (Amc), cefotaxime (Ctx), chloramphenicol (C), ciprofloxacin (Cip), gentamicin (G), trimethoprim (Tmp) and trimethoprim plus sulfamethoxazole (Sxt), and Clinical and Laboratory Standards Institute’s (CLSI) criteria [15] for kanamycin (Kan), nalidixic acid (Nal), streptomycin (Str), sulphonamides (Su) and tetracycline (T). Quality control was performed using the *Escherichia coli* ATCC 25922 strain. Multidrug resistance (MDR) was defined as resistance to 4 or more antibiotics.

### Multiple-locus variable-number of tandem repeats analysis (MLVA)

MLVA was performed as described previously [11]. Liquid cultures were heated at 95°C for 10 minutes and used directly in the PCR reaction after a brief centrifugation at 18,188 g for 10 minutes, a DNA lysate was prepared by heating a single colony in 300 μl sterile water at 100°C for 10 minutes and collecting the supernatant after centrifugation at 9,300 g for 10 minutes. PCR products were subjected to capillary electrophoresis on a ABI 3130xl Genetic Analyzer (Life Technologies), after which the size of the PCR products was determined with GeneMapper software v.1.0 (Life Technologies). GeneScan 600 LIZ (Life Technologies) was used as size standard. The calibration strains were the same as described previously [11]. MLVA profiles are reported as a string of 5 numbers (SENTR7-SENTR5-SENTR6-SENTR4-SE-3) representing the number of repeats at the corresponding locus. A frequent MLVA profile was defined as a MLVA profile that was detected in at least 35 isolates during the 6-year period 2007–2012 (with at least one isolate each year). A MLVA profile that was detected in less than 30 isolates during the 6-year period 2007–2012 defined a rare profile.

### Stability experiment

The *in vitro* stability of the 5 MLVA loci was evaluated in 20 *S*. Enteritidis isolates with a frequent MLVA profile and a frequent phage type, in 2 *S*. Enteritidis isolates with a rare MLVA profile but with a frequent phage type and in 5 *S*. Enteritidis isolates from different outbreaks in the Democratic republic of the Congo (DRC) between 2010 and 2011. A single colony from a culture grown overnight on LB agar at 37°C was inoculated into 5 ml LB broth and incubated overnight at 37°C without shaking. Next, a series of 50 passages at a rate of two passages per day was performed by inoculating 20 μl of culture into 5 ml fresh LB broth and incubating at 37°C without shaking. Glycerol (25% v/v) stocks (-80°C) were made before each 5th passage. MLVA was performed on heated liquid cultures after every fifth passage, as described above, leading to a total of 363 typing tests [16].

### Minimum spanning tree and diversity indices

A minimum spanning tree based on MLVA profiles of *S*. Enteritidis isolates was created in BioNumerics 6.5 (Applied Maths, Sint-Martens-Latem, Belgium) using the categorical coefficient and no priority rules for the algorithm.

The discriminatory power of phage typing, antibiotic susceptibility testing and MLVA was evaluated in the 1,498 randomly sampled *S*. Enteritidis isolates with Simpson’s index of diversity (D) [17] and Shannon’s indices of diversity (H’) and equitability (E) [18]. Shannon’s indices were calculated with the Biodiversity Calculator developed by J. Danoff-Burg and C. Xu [19].

## Results

### Phage types

**Fig 1:**
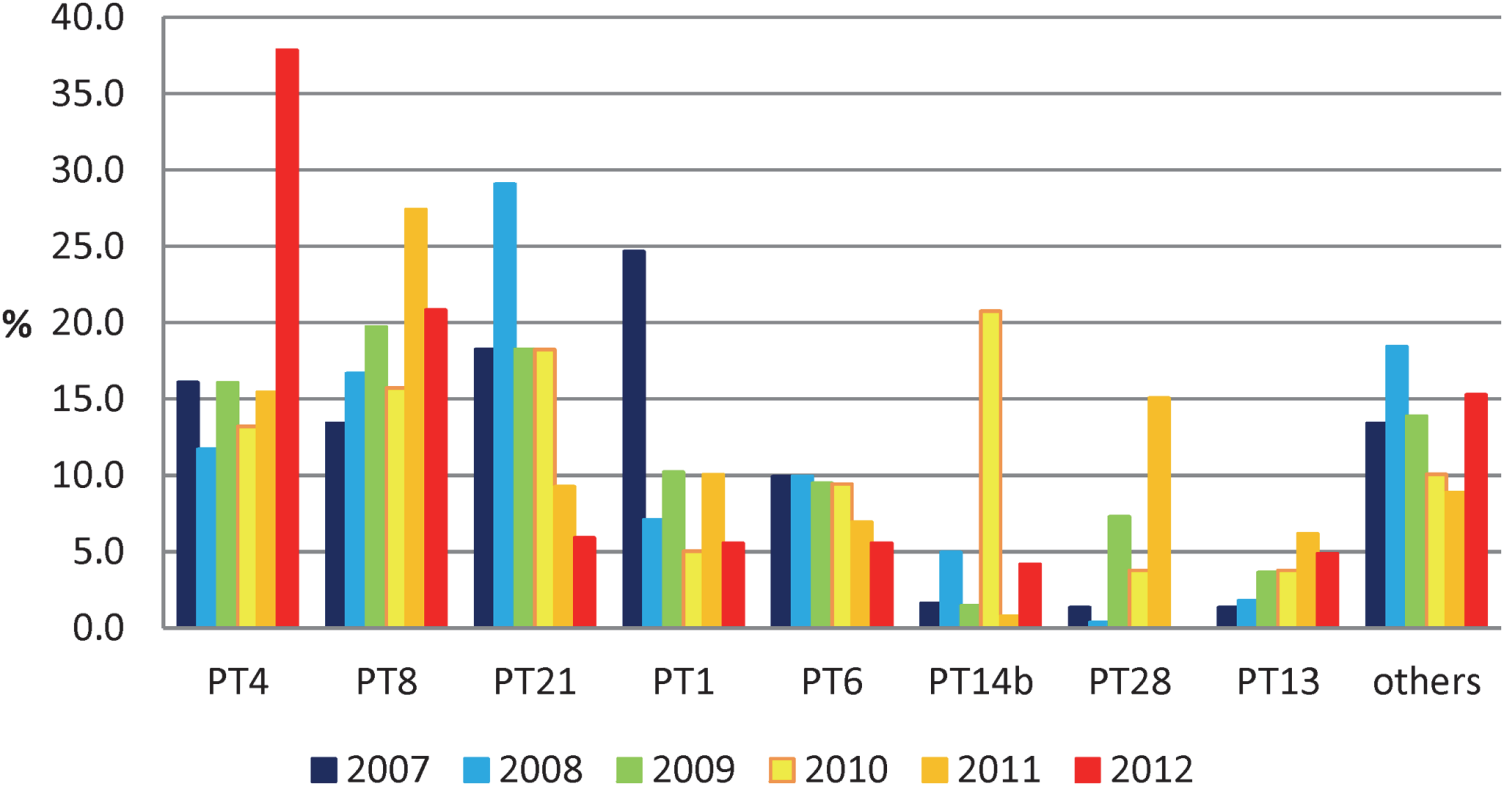
Phage type distribution among the S. Enteritidis population over the 6-year period (n- = 1,498; other: all phage types with less than 3% occurrence in the same combined population.

In the sample population of Belgian human isolates (1,498 isolates), 42 distinct phage types were present, nevertheless 1294 (86.4% of the sample population) isolates were attributed to the frequent phage types PT4 (19.0%), PT8 (18.6%), PT21 (16.3%), PT1 (11.7%), PT6 (8.5%), PT14b (4.6%), PT28 (4.1%) and PT13 (3.4%). The isolates with frequent phage types were not equally distributed over time (Fig. 1). Phage types PT1 and PT14b were mainly found in 2007 and 2010, respectively. Since the systematic vaccination of the layer flocks started in 2005, a decrease of PT4 and PT21 was observed from 23.6% in 2005 (data not shown) to 16.1% in 2007 (PT4) and from 21.0% in 2005 (data not shown) to 5.9% in 2012 for PT21.

The PT4 prevalence remained fairly stable until 2012 when a drastic and statistically significant (P < 0.001) increase was noted with a peak of 37.8%. The proportions of phage types PT28 and PT13 equally increased during the 6-year period. PT28 peaked in 2011 with a prevalence of 15.1% and this peak differed significantly from the values of the other years (P values varying between 0.05>P>0.02 and P<0.001). On the other hand, PT6 remained stable until 2010 when a significantly decreased to 5.6% in 2012 was noted (0.05>p>0.02). Both PT1 and PT21 decreased significantly (P<0.001) during the study period showing a 2012 prevalence of 5.6% and 5.9% respectively. In 2010, PT14b showed a unique peak of 20.8% which was significant different (P<0.001) from the values found in the other years. Other phage types comprised each less than 3% of the isolate panel.

### Antimicrobial susceptibility testing

Overall, the isolates of *S*. Enteritidis were generally susceptible to all antibiotics tested (87.0%).

Eight point five percent % of isolates were resistant to nalidixic acid and 3.3% to ampicillin. Proportion of Nalidixic acid and ampicillin resistant isolates varied between 5.3% and 13.7% and 1.25% and 5.04%, respectively over the 6-year period.

*S*. Enteritidis belonging to PT1 and PT14b showed resistance to nalidixic acid in 20.5% and 21.7% of the cases respectively.

Prevalence of resistance to other antibiotics was between 0.1% for ciprofloxacin, 0.7% for cefotaxime and 1.4% for sulfamethoxazole (Table 1).

**Table 1:** Overview of antimicrobial resistance and MLVA characteristics in relation to the phage type (n = 1,498).

Phage type	Nb. isolates (% of total)	Nb. susceptible isolates (%)	Nb. MDR isolates (%)	Most common resistance patterns (% of isolates with resistance pattern)	Nb. MLVA types	Most common MLVA profiles (SENTR7-SENTR5-SENTR6-SENTR4-SE-3) (% of isolates with MLVA profile)
PT4	284 (19.0)	253 (89,1)	4 (1.4)	Amp (1.1), Nal (8.5)	51	3–10–5–4–1 (38.2),3–11–5–4–1 (10.9)
PT8	279 (18.6)	274 (98.2)	1 (0.4)	Nal (0.4), Ctx (0.4), AmpCtxTetTmpSpeNalStrSulSxt (0.4)	40	2–10–7–3–2 (35.5), 2–11–7–3–2 (14.3)
PT21	245 (16.3)	235 (95.9)	3 (1.2)	Nal (2.0)	24	3–10–5–4–1 (27.7), 3–11–5–4–1 (27.3)
PT1	176 (11.7)	137 (77.8)	1 (0.6)	Nal (20.5), AmpAmxNal (1.1)	28	3–10–5–4–1 (68.6)
PT6	127 (8.5)	120 (94.5)	1 (0.8)	Amp (10.2), Nal (3.9)	24	3–10–5–4–1 (36.2), 3–9–5–4–1 (12.6), 3–10–5–3–1 (11.0)
PT14b	69 (4.6)	53 (76.8)	1 (1.5)	Nal (21.7)	15	2–13–7–3–2 (34.8), 3–10–5–4–1 (17.4)
PT28	61 (4.1)	61 (100)	0 (0)	-	21	2–10–7–3–2 (44.3)
PT13	51 (3.4)	27 (52.9)	0 (0)	Nal (9.8)	27	2–10–7–3–2 (15.7), 3–10–5–4–1 (7.8), 2–10–8–5–2 (7.8)
Other	206 (13.7)	162 (78.6)	8 (3.9)	Nal (8.7), AmpTet (1.9)	67	3–10–5–4–1 (17.9), 3–11–5–4–1 (9.2), 3–9–5–4–1 (7.7)

MDR: multidrug resistant; MLVA: multiple-locus variable-number of tandem repeats analysis; Other: phage types with less than 3% occurrence in the isolate panel; Amp: ampicillin; Amx: amoxicillin plus clavulanic acid; Chl: chloramphenicol; Nal: nalidixic acid; Str: streptomycin; Sul: sulphonamides; Sxt: trimethoprim plus sulfamethoxazole, Tet: tetracycline; Tmp: trimethoprim

Multi-resistance to at least ≥ 4 antibiotics was quite uncommon across all *S*. Enteritidis (1.3%).

### MLVA typing

Among the 1,498 *S*. Enteritidis isolates typed with MLVA targeting 5 loci, a total of 172 distinct MLVA profiles were detected. The highest number of different alleles was seen at locus SENTR5 (15), followed by loci SENTR6 (12), SENTR4 (7), SENTR7 (4) and SE3 (2).

**Fig 2:**
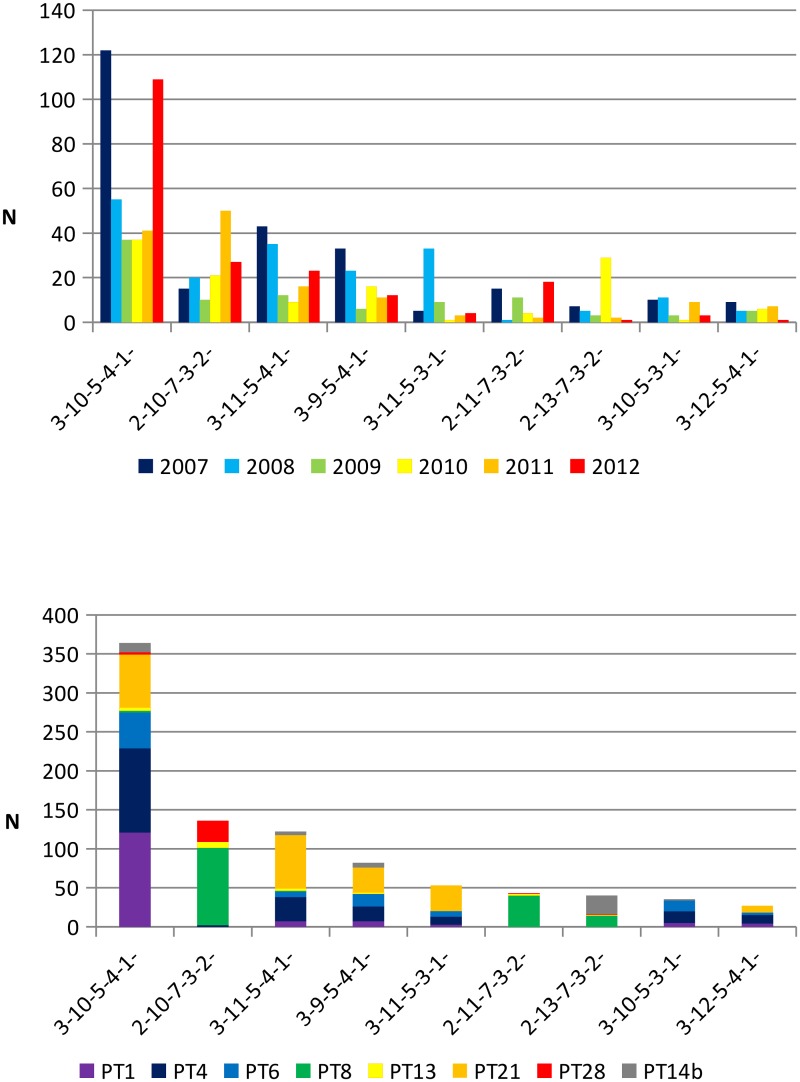
a: Annual distribution of Belgian S. Enteritidis isolates over the 6-year period according to the nine most frequent MLVA profiles (representing 1,006 of the 1,498; 67.2%). b: Prevalence of *S*. Enteritidis isolates in this study with the most frequent MLVA profile and with indication of the major phage type (representing 902 of the 1,498; 60.2%).

Eigthy nine rare MLVA profiles (51.7% of the MLVA profiles) were detected in only one *S*. Enteritidis isolate (5.9% of the sample population), while 9 frequent MLVA profiles (5.2% of the MLVA profiles) comprised 67.2% of the *S*. Enteritidis isolates (Fig. 2a and 2b). The MLVA profile 3–10–5–4–1 was the most common profile detected throughout the 6-year period and represented 26.7% of the *S*. Enteritidis isolates (Fig. 2b).

The isolates with frequent MLVA profile were not equally distributed over the years of the study period (Fig. 2a). Profiles 3–11–5–3–1 and 2–13–7–3–2- were mainly found in 2008 and 2010. Profile 3–10–5–4–1 was dominant in 2007 and 2012 (Fig. 2a).

Among the most frequent phage types, the number of distinct MLVA profiles ranged from 15 for PT14b and to 51 for PT4 (Table 1).

**Fig 3:**
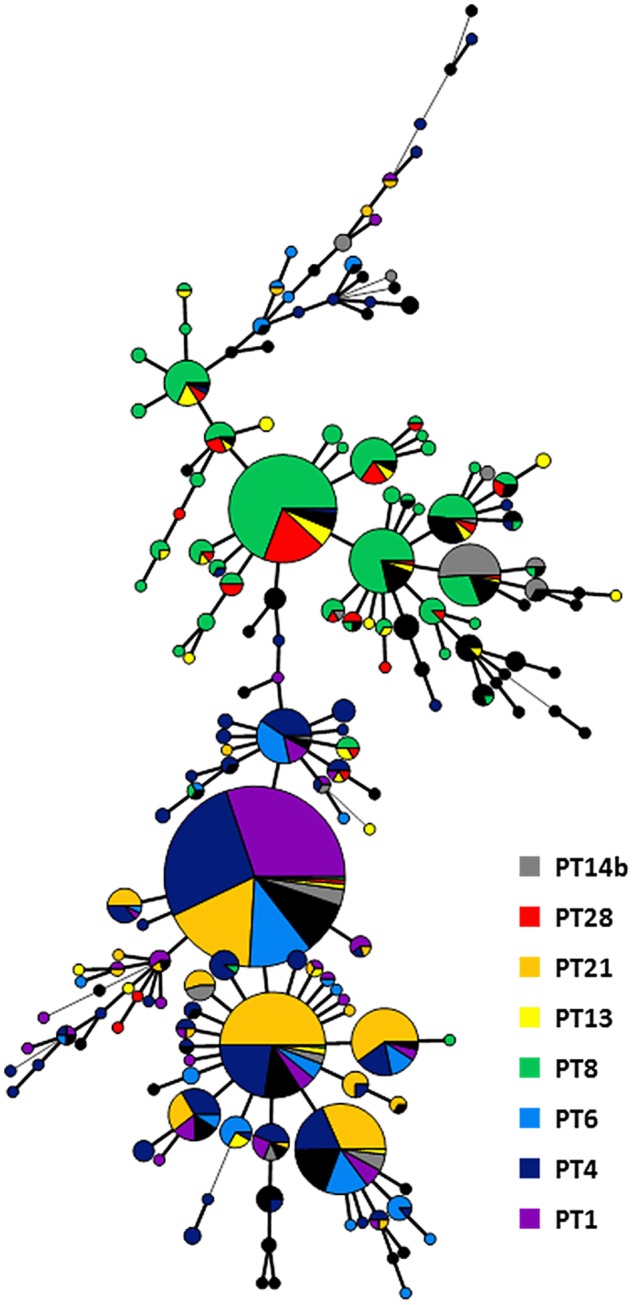
Minimal spanning tree calculated of MLVA profiles of S. Enteritidis isolates over the 6-year period. Each node represents a different MLVA profile with frequency-dependent size. Node colour represents phage type: Branch thickness indicates how many loci are different in the MLVA profiles of the connected nodes. Thick solid lines connect nodes that differ by one MLVA locus, thin solid lines connect nodes that differ by two MLVA loci and dashed lines connect nodes that differ by three MLVA loci.

Except for phage type PT13 and PT21, which showed a dispersed population of many different MLVA profiles, the most common MLVA profile for each phage type belonged to one of the frequent MLVA profile (Table 1, Fig. 2b). The major profile 3–10–5–4–1 was common in the isolates belonging to PT1, PT4 and PT6. Isolates belonging to PT8 mostly presented the profiles 2–10–7–3–2 and 2–11–7–3–2; PT28 isolates mostly presented the profile 2–10–7–3–2. On the other hand, the isolates belonging to PT21 were almost equally dispersed in four different profiles 3–10–5–4–1, 3–11–5–4–1, 3–9–5–4–1 or 3–11–5–3–1 (Fig. 2b, Fig. 3).

### Diversity of phage typing, antimicrobial susceptibility testing and MLVA

Diversity indices are commonly used as a measure for the discriminatory power, defining the ability to distinguish between unrelated isolates [17], of a subtyping method. The higher the value of a diversity index, the higher the discriminatory power of the subtyping method. Simpson’s D ranges from 0 to 1 and gives the probability that 2 randomly sampled and unrelated isolates will have a different subtype [17]. Rare subtypes, which apply to only a small number of isolates, will have a small contribution to the Simpson’s index and as such, the number of subtypes has little influence on this index [20]. Shannon’s H’ is an indicator for subtype richness [20] and its highest value is ^e^log(S), where S is the number of subtypes. Shannon’s E is a measure for the evenness of the subtype distribution [20] and has 1 as maximum value. Calculated values for Simpson’s D, Shannon’s H’ and E indices were, respectively, 0.88, 2.45 and 0.66 for phage typing, 0.61, 1.51 and 0.40 for antimicrobial susceptibility testing, and 0.90, 3.36 and 0.65 for MLVA. The combination of MLVA-profile and phagetype resulted in a Simpson’s D of 0.97, a Shannon’s H’ of 5.09 and E of 0.87. These values suggest that MLVA and phagetyping have similar discriminatory power, which is significantly higher than that of antimicrobial susceptibility testing. A combination of both MLVA and phagetyping further improves the discriminatory power and the evenness of the subtype distribution.

### Typing of outbreak isolates

To evaluate the added value of MLVA in case of outbreak detection, a subset of 37 isolates representative for sampling date and geography originating from different outbreaks in the Democratic republic of the Congo (DRC) between 2010 and 2011 were included in this study.

MLVA typing of the 37 isolates revealed two major profiles: 2–13–3–3-NA (n = 16) and 2–15–3–3-NA (n = 14). In addition, there were three minor profiles: 2–14–3–3-NA and 2–16–3–3-NA (4 and 2 isolates respectively, from patients of two neighboring health centers, Nkandu and Kikonka) and profile 1–15–3–3–1 (1 isolate from a patient in Ngeba).

The respective MLVA profiles were distinct from any of the MLVA profiles found in Belgian isolates throughout the six year observational period. The differences between 5 of the 6 detected MLVA profiles were minor and can be attributed to one variation of tandem repeat number in only one locus (SENTR5).

### Stability of MLVA loci

To evaluate the *in vitro* stability of the number of tandem repeats in the MLVA loci, 27 *S*. Enteritidis isolates (22 isolates from Belgium and 5 from DRC) were subjected to a serial passage experiment (a series of 50 passages at a rate of two passages per day see Materials and Methods). The 22 Belgian isolates selected for this stability experiment covered all frequent phage types and MLVA profiles (Table 2). Five Isolates from the Congo outbreak were also selected in function of their frequency (Table 3). Among the 27 isolates, no variation in the MLVA profiles could be detected throughout the serial passage experiment indicating that all the studied MLVA profiles were stable.

**Table 2:** Overview of isolates and outcome of the stability experiment for the Belgian isolates(n = 22).

ID Number	Phage Type	Initial MLVA profile	Final MLVA profile	Frequence of the Phage type and MLVA profile (%/%)
SE1: 07–1456	PT8	2–13–7–3–2	2–13–7–3–2	18.6/3.1
SE2: 07–5973	PT28	2–12–7–3–2	2–12–7–3–2	4.1/0.01
SE3: 09–2821	PT8	2–13–7–3–2	2–13–7–3–2	18.6/3.1
SE4: 11–2950	PT28	2–10–7–3–2	2–10–7–3–2	4.1/9.5
SE5: 12–0215	PT14b	2–13–7–3–2	2–13–7–3–2	4.6/3.1
SE6: 12–0266	PT4	3–9–5–4–1	3–9–5–4–1	19/6.7
SE7: 12–0429	PT21	3–11–5–4–1	3–11–5–4–1	16.3/9.2
SE8: 12–1442	PT21	3–11–5–4–1	3–11–5–4–1	16.3/9.2
SE9: 12–2353	P13	2–12–7–3–2	2–12–7–3–2	3.4/0.01
SE10: 12–2488	PT21	3–9–5–4–1	3–9–5–4–1	16.3/6.7
SE11: 12–2592	PT14b	3–10–5–4–1	3–10–5–4–1	4.6/26.8
SE12: 12–2630	PT21	3–10–5–4–1	3–10–5–4–1	16.3/26.8
SE13: 12–3578	PT1	3–10–5–4–1	3–10–5–4–1	11.7/26.8
SE14: 12–3599	PT4	3–11–5–4–1	3–11–5–4–1	19/9.2
SE15: 12–3693	PT4	3–10–5–4–1	3–10–5–4–1	19/26.8
SE16: 12–3723	PT8	2–10–7–3–2	2–10–7–3–2	18.6/9.5
SE17: 12–4071	PT8	2–11–7–2–2	2–11–7–2–2	18.6/3.4
SE18: 12–4164	PT8	2–11–7–2–2	2–11–7–2–2	18.6/3.4
SE19: 12–4561	PT8	2–11–7–3–2	2–11–7–3–2	18.6/3.4
SE20: 12–4593	PT4	3–10–5–3–1	3–10–5–3–1	19/2.4
SE21: 12–4666	PT6	3–10–5–3–1	3–10–5–3–1	8.5/2.4
SE22: 12–4775	PT4	3–11–5–3–1	3–11–5–3–1	19/3.6

**Table 3:** Overview of isolates and outcome of the stability experiment for the outbreak isolates from Congo (n = 5).

ID Number	Initial MLVA profile	Final MLVA profile	Frequence of the MLVA profile (n/37)
SE23: 3303–3 Congo	2–16–3–3-NA	2–16–3–3-NA	2/37
SE24: 3339–3 Congo	2–15–3–3-NA	2–15–3–3-NA	14/37
SE25: 3493–3 Congo	2–15–3–3-NA	2–15–3–3-NA	14/37
SE26: 3522 Congo	2–14–3–3-NA	2–14–3–3-NA	4/37
SE27: 3680 Congo	2–14–3–3-NA	2–14–3–3-NA	4/37

## Discussion

In the EU, *S*. Enteritidis and *S*. Typhimurium are most frequently associated with human salmonellosis [1]. The most common serovar of *Salmonella* isolated from human outbreaks is Enteritidis [1]. In Belgium, since the drastic decrease observed in 2005 [3], Enteritidis is the second most prevalent serovar isolated from human patients after Typhimurium. Hence subtyping of this serovar is very important for outbreak detection and tracing outbreak sources.

The Belgian NRCSS relies on phage typing and antimicrobial susceptibility testing for routine surveillance of *S*. Enteritidis, complemented with PFGE during outbreak investigations. PFGE, which is widely considered as the gold standard for subtyping of *Salmonella*, is a labour intensive and time consuming technique and therefore implementation of this subtyping method for routine surveillance is not feasible for the Belgian NRCC [9]. MLVA, which requires less hands-on time and allows faster typing and easy inter-laboratory comparison of results, has been adopted by several European countries for surveillance and detection and investigation of outbreaks caused by *S*. Typhimurium [21]. Nevertheless, for *S*. Enteritidis, the MLVA typing method is still under evaluation [11,22]. A harmonised protocol that can be used as gold standard in Europe has been developed by Hopkins in 2011[11]. The present study assessed the capability of MLVA typing for surveillance and outbreak detection of human *S*. Enteritidis in Belgium, 1,498 isolates collected over the 6-year period 2007–2012 were characterised by phage typing, antimicrobial susceptibility testing and MLVA.

Our study showed that phage types PT4, PT8, PT21, PT1, PT6, PT14b, PT28 and PT13 dominated the *S*. Enteritidis population in Belgium over the six year observation period. Some fluctuations in prevalence of phage types were observed over time: PT1 predominated in 2007, PT21 in 2008, PT14b in 2010, PT8 in 2011 and PT4 in 2012. Like in other countries; these fluctuations are probably mainly due to outbreaks [23,24], [25] even if the sources of contamination could not always be identified [23,24]. The prevalence of PT28 increased over the six-year continuously. In Belgium a shift in the distribution of phage types was observed between the period of the implementation of layer flock vaccination (2003–2005) and after (2007–2012)[5]. PT4 and PT21, the two main PT, represented all together between about 65% in 2003–2005 and 35% in 2007–2012 of all the *S*. Enteritidis.

Nevertheless, the eight most common PTs accounted for approximately 86.3% of all sub-typed strains of *S*. Enteritidis and each year one phage type represented more than 20% of the typed strains. This predominance of a small number of phage types was also observed in other countries and reduces the capacity of phage typing as a discriminating tool to investigate outbreak isolates.

Antimicrobial susceptibility testing is another subtyping method used in public health surveillance. In our study,*S*. Enteritidis isolates were, in majority, pan-susceptible to antimicrobials. Our study showed that Nalidixic acid resistant PT14b and PT1 *S*. Enteritidis isolates are circulating in Belgium as has been reported in other European countries too [24]. Low rates of resistance and MDR detected in *S*. Enteritidis are in agreement with other studies that have reported a low prevalence of antimicrobial resistance among *S*. Enteritidis isolated from different sources [26,27]. For this reason, antimicrobial susceptibility testing is not always sufficiently discriminating in the surveillance of outbreaks caused by *S*. Enteritidis.

Molecular techniques like MLVA are generally considered to improve surveillance and detection of outbreaks and their sources [21,23]. The possibility to present the result as a string of five numbers is one of the strengths of MLVA and allows easy international sharing across country borders and setup of databases of MLVA profiles [11]. Yet, laboratories have to agree on the set of calibration strains and on the nomenclature used, so that laboratories report standardized MLVA profiles [11,22]. Another already reported asset of MLVA subtyping is the high discriminatory power of this technology. In our study, MLVA divided the investigated *S*. Enteritidis collection into 172 distinct profiles and indeed allowed for discrimination within isolates of the same phage type, which is in concordance with previous studies. Most of the MLVA profiles have already been described in other European countries [11]. In order to compare the discriminatory power in an objective manner, diversity indices were calculated for the different subtyping methods used in this study. A similar Simpson’s index of diversity (D) was obtained for MLVA and phage typing, which were higher than for antimicrobial susceptibility testing. This demonstrates the small influence of the number of subtypes on Simpson’s diversity index, as MLVA testing revealed 172 distinct patterns compared to only 42 detected phage types, while resulting in equal discriminatory power according to Simpson’s index. Shannon’s diversity (H’) and equitability (E) indices, which are indicators of the number of subtypes and of the evenness of the distribution of these subtypes [20], respectively, offer a more differentiated measure for comparison of discriminatory power, i.e. aiming at a high number of evenly distributed subtypes. Shannon’s H’ denotes that MLVA testing was slightly more able to discriminate between unrelated isolates than phage typing, but Shannon’s E values indicate that types are equally distributed for both MLVA testing and phage typing.

The nine most frequent MLVA profiles comprised 67.2% of all *S*. Enteritidis isolates collected in Belgium and the most prevalent MLVA profile represented 26.7% of them. The inability of the MLVA method to discriminate different isolates in this dominant group is the major disadvantage of this technique.

The addition of four more loci as described by Malorny et al. ([10])and Hopkins et al ([11]) did not result in a further discrimination (data not shown).

Our study showed that isolates belonging to the prevalent MLVA profile group can be subdivided to 5 common phage types (PT1, PT4, PT6, PT21 and PT14b) proving that a better discrimination was achieved by combining the phage typing method and MLVA. This is also demonstrated by the diversity and distribution indices. On the contrary, MLVA or phage typing might be sufficient to distinguish a cluster of isolates with rare MLVA profiles or phage types. This was also the case for the 37 outbreak isolates presented in this study.

In addition to the discriminatory power of a subtyping method, the stability of the assessed markers should be taken into account. From our serial passage experiment on *S*. Enteritidis isolates, we observed that all the MLVA profiles remained stable *in vitro* proving the stability of the results obtained by this technique.

In conclusion, based on Simpson’s and Shannon’s indices, MLVA has a high discriminatory power for the 1,498 *S*. Enteritidis collected during the 6-year period 2007–2012, and can thus improve public health surveillance. However, outbreak detection with MLVA is not straightforward, since for isolates with a frequent MLVA profile, phage typing is still necessary to achieve an unique, combined subtyping result. MLVA should be complemented with other subtyping methods when the investigated outbreak is caused by the most common MLVA profile.
