# Correction: Multi Locus Variable-Number Tandem Repeat (MLVA) Typing Tools Improved the Surveillance of *Salmonella* Enteritidis: A 6 Years Retrospective Study

**DOI:** 10.1371/journal.pone.0198527

**Published:** 2018-05-30

**Authors:** Sophie Bertrand, Guillaume De Lamine de Bex, Christa Wildemauwe, Octavie Lunguya, Marie France Phoba, Benedikt Ley, Jan Jacobs, Raymond Vanhoof, Wesley Mattheus

Prior to the publication of this *PLOS ONE* article [1], the authors published a closely related work also in *PLOS ONE* [2]. The two MLVA studies were performed in the Belgian National Reference Centre for Salmonellas (NRC) at the same time, following the same schema and using the same experimental methodology. The data of the current study were presented in the same way as the first MLVA study published in 2013 [2] to give readers the opportunity to compare the added value of the MLVA analysis on the two most prevalent Salmonella serotypes (Enteritidis and Typhimurium). The first publication was cited in the current publication; however, the authors recognize that it should have been more clearly discussed in the current publication.

The authors also acknowledge the reuse of text between the two publications. Specifically, the reuse of text concerns the following parts:

- Abstract: The introductory sentence and the methodology used to evaluate the stability of the MLVA profiles.
- Materials and Methods
- Results: The methodology of calculation to evaluate the diversity of phage typing, antimicrobial susceptibility testing and MLVA.
- Discussion: Description of the current subtyping methodology for routine surveillance of Salmonella and comparison with MLVA; repeating the main conclusions of the earlier study
